# Molecular landscape and clinical significance of exon 11 mutations in KIT gene among patients with gastrointestinal stromal tumor: a retrospective exploratory study

**DOI:** 10.3389/fonc.2023.1272046

**Published:** 2023-10-12

**Authors:** Ruihua Zhao, Tianqi An, Min Liu, Yanan Zhou, Rui Li, Guozhong Jiang, Jing Li, Xinguang Cao, Hong Zong

**Affiliations:** ^1^ Department of Oncology, The First Affiliated Hospital of Zhengzhou University, Zhengzhou, China; ^2^ Department of Medical Science, Berry Oncology Corporation, Beijing, China; ^3^ Department of Endoscopy Center, Anyang Cancer Hospital, Anyang, China; ^4^ Department of Oncology, Tongji Hospital, Huazhong University of Science and Technology, Wuhan, China; ^5^ Department of Pathology, The First Affiliated Hospital of Zhengzhou University, Zhengzhou, China; ^6^ Department of Digestive Disease, The First Affiliated Hospital of Zhengzhou University, Zhengzhou, China

**Keywords:** gastrointestinal stromal tumors, KIT exon 11, mutation, sanger sequencing, prognosis

## Abstract

**Objective:**

This aim of this study was to investigate the prognostic significance of KIT exon 11 mutation subtypes in patients with GISTs.

**Methods:**

A total of 233 consecutive patients diagnosed with GISTs at the First Affiliated Hospital of Zhengzhou University from January 2013 to August 2018 were included in this study. The prevalence and mutation landscape of exon 11 in KIT was presented. The clinicopathological characteristics and prognosis among the different mutation subtypes were analyzed. All the statistical analyses were performed by SPSS22.0.

**Results:**

Somatic mutational analysis indicated that point mutations were the most frequently detected mutations followed by deletions & compound mutations and insertion and tandem duplication mutations in the stomach. Point mutations showed a low mitotic count and a high risk of recurrence, and deletions and compound mutations have a high mitotic count while insertions and tandem duplication mutations showed a low mitotic count with an intermediate recurrence risk. Point mutations and deletions frequently occurred in sequence region codons 550-560 of exon 11, while compound mutations, insertion, and tandem duplication were mainly detected in codons 557-559, 572-580, and 577-581, respectively. The multi-variation analysis demonstrated that tumor diameter and high recurrence risk groups had worse prognostic values. However, mutation types were not significant predictors of relapse-free survival (RFS) in GISTs. Survival analysis suggested no significant difference in RFS between the 557/558 deletion and the other deletions.

**Conclusion:**

This study suggested that mutations in exon 11 of the KIT gene were common with intermediate/high recurrence risk in GISTs patients. Tumor diameter ≥5 cm, and deletions mutations might predict a worse prognosis.

## Introduction

Gastrointestinal stromal tumors (GISTs) are the most common mesenchymal tumors of the gastrointestinal tract, characterized by functional mutations in KIT gene or platelet-derived growth factor receptor α (PDGFRA) (1), approximately 20,000 new cases of GIST are annually diagnosed in China (2). Relevant medical evidence showed a long-term prognosis for patients with these tumors own to the rarity of GISTs currently (3). Although surgery remained the primary therapeutic option for patients with advanced disease, the prognosis after resection of GISTs is enormously disappointing (4). Even though various prognostic criteria were developed in the past few decades to stratify the risk factors for patients treated with curative resection, none of these criteria considered tumor mutation status for the risk stratification (5). Imatinib was the dominating targeted drug for patients with GISTs in both early and advanced stages. Risk of recurrence existed in GIST even if patients underwent complete resection. Therefore, adjuvant imatinib therapy was recommended for GIST patients with intermediate and high risk according to the modified National Institutes of Health (NIH) classification currently (6). Still and all, more factors should be considered for risk stratification to improve the prognosis of patients subsequently.

KIT gene is located in chromosome 11p13 and contains 21 exons, which is a type 3 transmembrane receptor for mast cell growth factor, also known as stem cell factor, and the mutations in this gene are associated with gastrointestinal stromal tumors, mast cell disease, acute myelogenous leukemia, and piebaldism (7). The KIT mutations were used to predict imatinib efficacy in GISTs and as a valuable prognostic predictor in GISTs (8). A previous study indicated that the overall prevalence of KIT mutation among patients with GIST was approximately 75%. The common mutation types in KIT were exon 9 (8%), exon 11, exon 13 (1%), and exon 17 (1%), with exon 11 of the highest frequency (65%) (9). Concerning the prognostic significance of KIT mutation, the previous study indicated that the prognosis was worse among patients with mutation-positive GISTs than those with mutation-negative GISTs (10).

Additionally, the subsequent work suggested that patients with exon 9 mutations conferred a higher risk of progression than those with exon 11 (11). And exon 11 mutations were usually divided into five different subtypes (deletions, point mutations, compound mutations, insertions, and tandem duplication mutations) (12). Joensuu et al. concluded that patients with KIT exon 11 deletions or duplication mutation were more likely to confer favorable RFS (13). However, Jiang et al. indicated that patients with KIT exon 11 deletion had a lower 5-year PFS rate than other KIT exon 11 mutations (14). Furthermore, exon 11 mutation subtypes of GIST were concluded by meta-analysis or systematic literature review on the rarity of GIST, resulting in the insufficiency of the clinical evidence for KIT exon 11 mutations, especially in the Chinese population (15). As a result, different mutation subtypes in exon 11 might confer distinct prognosis in patients with GIST. However, there still needed to be a consensus regarding their prognostic significance.

Consequently, the primary objective of our study was to investigate the clinicopathological characteristics of GISTs with KIT exon 11 mutations and to identify the prognostic significance of KIT mutation in a large-scale cohort of Chinese patients with GIST retrospectively.

## Patients and methods

### Design of the study and eligible patients

A total of 233 patients diagnosed with GIST in the First Affiliated Hospital of Zhengzhou University from January 2013 to August 2018 were included in this study consecutively and retrospectively. The GIST patients fulfilling the following criteria were included (1): histologically and pathologically confirmed GIST (2); Eastern cooperative oncology group (ECOG) performance status of 0-2 score (3); patients with GIST were treated with complete surgical resection in clinical practice. In contrast, patients having (1) double KIT exon mutations (2); extremely low risk (3); previous exposure to radiotherapy or chemotherapy during initial treatment; (4) familial GISTs; (5) diagnosis of other primary tumors before or after the diagnosis of GISTs; (6) severe cardiovascular or mental illness; (7) survival data of the patients were not available were excluded from the study.

The detailed clinicopathological characteristics of the recruited patients are presented in **Table 1**. The onset of diagnosis and recurrence were determined by pathological diagnosis and radiographic examination, respectively. Recurrence risk was determined according to the NIH modified grading criteria (16). Patients with radical surgical resection were examined by CT and gastrointestinal endoscopes every 3-6 months. The baseline characteristics and survival data were mainly acquired from our hospital’s electronic medical record system and interviewing patients. The Clinical Research Ethics Committee of the First Hospital of Zhengzhou University (approved number: 2021-KY-1080-002) has approved the sampling and experimental procedures of this study. Informed written consent was obtained from all the patients before the commencement of the study, and we strictly followed the recommendations of the Declaration of Helsinki 1964.

**Table 1:** The clinicopathological characteristics of 233 patients with GISTs.

Characteristics	Point mutations	Deletions	Compound mutation	Insertions	Tandem duplications	Total
	(N=84, %)	(N=78, %)	(N=55, %)	(N=10, %)	(N=6, %)	(N=233, %)
Gender						
Male	32 (38.1)	38 (48.7)	26 (47.3)	5 (50.0)	2 (33.3)	103 (44.2)
Female	52 (61.9)	40 (51.3)	29 (52.7)	5 (50.0)	4 (66.7)	130 (55.8)
Age						
<60	38 (45.2)	49 (62.8)	31 (56.4)	7 (70.0)	3 (50.0)	128 (54.9)
≥60	46 (54.8)	29 (37.2)	24 (43.6)	3 (30.0)	3 (50.0)	105 (45.1)
tumor location						
Stomach	55 (65.5)	44 (56.4)	24 (43.6)	10 (100.0)	4 (66.6)	137 (58.8)
Intestines	26 (30.9)	29 (37.2)	23 (41.8)	0 (0.0)	1 (16.7)	79 (33.9)
Other*	3 (3.6)	5 (6.4)	8 (14.6)	0 (0.0)	1 (16.7)	17 (7.3)
tumor diameter (cm)						
≤2	3 (3.6)	4 (5.1)	1 (1.8)	1 (10.0)	0 (0.0)	9 (3.9)
2-5	31 (36.9)	30 (38.5)	18 (32.7)	3 (30.0)	2 (33.3)	84 (36.0)
5-10	42 (50.0)	33 (42.3)	24 (43.7)	4 (40.0)	3 (50.0)	106 (45.5)
>10	8 (9.5)	11 (14.1)	12 (21.8)	2 (20.0)	1 (16.7)	34 (14.6)
Mitotic count (/50HPF)						
≤5	49 (58.3)	28 (35.9)	18 (32.7)	6 (60.0)	4 (66.7)	105 (45.1)
6-10	27 (32.2)	21 (26.9)	18 (32.7)	4 (40.0)	2 (33.3)	72 (30.9)
>10	8 (9.5)	29 (37.2)	19 (34.6)	0 (0.0)	0 (0.0)	56 (24.0)
Recurrence risk						
Low risk	15 (17.8)	10 (12.8)	9 (16.4)	1 (10.0)	1 (16.7)	36 (15.5)
Intermediate risk	33 (39.3)	19 (24.4)	10 (18.2)	7 (70.0)	3 (50.0)	72 (30.9)
High risk	36 (42.9)	49 (62.8)	36 (65.4)	2 (20.0)	2 (33.3)	125 (53.6)
CD117						
Negative	1 (1.2)	1 (1.3)	0 (0.0)	0 (0.0)	0 (0.0)	2 (0.9)
Positive	83 (98.8)	77 (98.7)	55 (100.0)	10 (100.0)	6 (100.0)	231 (99.1)
DOG-1						
Negative	2 (2.4)	3 (3.9)	1 (1.8)	1 (10.0)	0 (0.0)	7 (3.0)
Positive	82 (97.6)	75 (96.1)	54 (98.2)	9 (90.0)	6 (100.0)	226 (97.0)
CD34						
Negative	9 (10.7)	10 (12.8)	6 (10.9)	0 (0.0)	1 (16.7)	26 (11.2)
Positive	75 (89.3)	68 (87.2)	49 (89.1)	10 (100.0)	5 (83.3)	207 (88.8)
Ulcer						
No	77 (91.7)	74 (94.8)	51 (92.7)	9 (90.0)	6 (100)	217 (93.1)
Yes	11 (8.3)	4 (5.2)	4 (7.3)	1 (10.0)	0 (0.0)	16 (6.9)
Bleeding						
No	72 (85.7)	70 (89.7)	46 (83.6)	9 (90.0)	4 (66.7)	201 (86.3)
Yes	12 (14.3)	8 (10.3)	9 (16.4)	1 (10.0)	2 (33.3)	32 (13.7)
Necrosis						
No	74 (88.1)	64 (82.1)	46 (83.6)	7 (70.0)	5 (83.3)	196 (84.1)
Yes	10 (11.9)	14 (17.9)	9 (16.4)	3 (30.0)	1 (16.7)	37 (15.9)
Imatinib						
No	18 (21.4)	14 (17.9)	9 (16.4)	0 (0.0)	2 (33.3)	43 (19.3)
Yes	50 (59.5)	53 (67.9)	42 (76.4)	6 (0.6)	3 (50.0)	154 (69.1)
Unknown	16 (19.1)	11 (14.2)	4 (7.2)	4 (0.4)	1 (16.7)	36 (11.6)

*peritoneum, pelvic cavity, abdominal cavity, esophagus and adrenal gland.

### DNA sequencing

DNA was extracted from Formalin-fixed paraffin-embedded (FFPE) tissue using a DNA extraction kit (QIAamp DNA Mini Kit, Qiagen, Germany) following the manufacturer’s instructions. For each specimen, areas containing at least 50% tumor cells were selected from the hematoxylin-eosin-stained sections. Exon 11 of the KIT gene was amplified by Polymerase chain reaction (PCR) using a specific set of primers (forward primer, 5’-CCAGAGTGCTCTAATGACTG-3’; reverse primer, 5’-ACCCAAAAAGGTGACATGGA-3’) mentioned in previous study (17). Amplicons were sequenced using an ABI3500 Dx sequencer (Applied Biosystems Inc., Foster City, CA) and analyzed by Chromas software. The protocol for KIT mutation analysis was adopted from the previous study (18), and molecular pathologists confirmed the results. PCR amplification and sequencing were performed in duplicate for each sample.

### Statistically analysis

The study’s primary endpoint was recurrence-free survival (RFS), defined as the previous study (19). The statistical analysis was performed by SPSS 22.0. The correlation between mutation subgroups of exon 11 in the KIT gene and clinicopathological characteristics was assessed by the chi-square test. Cox univariate analysis was used to identify the association between clinicopathological characteristics and RFS. Furthermore, the survival curves were generated using Stata 14.0. And the statistical significance of the survival curves was produced using the log-rank test. A multivariate Cox regression analysis was constructed for multivariable analysis, and odds ratio (OR) and 95% confidence interval (95% CI) were computed. Statistical significance was accepted when *P*<0.05.

## Results

### Baseline characteristics

A total of 233 patients (103 males, 130 females, and age range 15-83 years old (median 60 years old)) with GISTs analyzed for KIT exon 11 mutations. The median tumor size was 6.0 cm (range 0.3-19 cm). Additionally, other detailed characteristics of the patients included are shown in **Table 1**.

### Correlation between KIT exon 11 mutations and baseline characteristics

The correlation between KIT exon 11 mutations and the baseline characteristics are shown in **Table 2**. Patients with the different mutation types in exon 11 showed significant association with age, tumor location, mitotic count and recurrence risk.

**Table 2:** The correlation between KIT exon 11 mutations in GIST patients with complete surgical resection and baseline characteristics.

Characteristics		GISTs with *KIT* exon 11 mutations								
		Point mutations n (%)	Deletion and compound mutations n (%)	*P*	Other mutations n (%)	Deletion and compound mutations n (%)	*P*	Point mutations n (%)	Other mutations n (%)	*P*
Gender	Male	32 (38.1)	64 (48.1)	0.148	7 (43.7)	64 (48.1)	0.796	32 (38.1)	7 (43.7)	0.781
	Female	52 (61.9)	69 (51.9)		9 (56.3)	69 (51.9)		52 (61.9)	9 (56.3)	
Age	<60	38 (45.2)	80 (60.2)	0.032^*^	10 (62.5)	80 (60.2)	0.856	38 (45.2)	10 (62.5)	0.277
	≥60	46 (54.8)	53(39.8)		6 (37.5)	53 (39.8)		46 (54.8)	6 (37.5)	
Tumor location	Stomach	55 (65.5)	68 (51.1)	0.038^*^	14 (87.5)	68 (51.1)	0.006^**^	55 (65.5)	14 (87.5)	0.138
	Others**^#^**	29 (44.5)	65 (48.9)		2 (12.5)	65 (48.9)		29 (44.5)	2 (12.5)	
Tumor diameter (cm)	≤5	34 (40.5)	53 (39.8)	0.927	6 (37.5)	53 (39.8)	0.856	34 (40.5)	6 (37.5)	>0.999
	>5	50 (59.5)	80 (60.2)		10 (62.5)	80 (60.2)		50 (59.5)	10 (62.5)	
Mitotic count (/50HPF)	≤5	49 (58.3)	46 (34.6)	0.001^***^	10 (62.5)	46 (34.6)	0.029^*^	49 (58.3)	10 (62.5)	0.79
	>5	35 (41.7)	87 (65.4)		6 (37.5)	87 (65.4)		35 (41.7)	6 (37.5)	
Recurrence risk	Low/Intermediate	48 (58.1)	48 (36.1)	0.003^**^	12 (75.0)	48 (36.1)	0.003^**^	48 (58.1)	12 (75.0)	0.266
	High	36 (42.9)	85 (63.9)		4 (25.0)	85 (63.9)		36 (42.9)	4 (25.0)	
CD117	Negative	1 (1.2)	1 (0.8)	0.742	0 (0)	1 (0.8)	>0.999	1 (1.2)	0 (0)	>0.999
	Positive	83 (98.8)	132 (99.2)		16 (100)	132 (99.2)		83 (98.8)	16 (100)	
DOG-1	Negative	2 (2.4)	4 (3.0)	>0.999	1 (6.2)	4 (3.0)	>0.999	2 (2.4)	1 (6.2)	0.411
	Positive	82 (97.6)	129 (97.0)		15 (93.8)	129 (97.0)		82 (97.6)	15 (93.8)	
CD34	Negative	9 (10.7)	16 (12.0)	0.767	1 (6.2)	16 (12.0)	0.696	9 (10.7)	1 (6.2)	>0.999
	Positive	75 (89.3)	117 (88.0)		15 (93.8)	117 (88.0)		75 (89.3)	15 (93.8)	
Ulceration	No	77 (91.7)	125 (94.0)	0.139	15 (93.8)	125 (94.0)	>0.999	77 (91.7)	15 (93.8)	>0.999
	Yes	7 (8.3)	8 (6.0)		1 (6.2)	8 (6.0)		7 (8.3)	1 (6.2)	
Bleeding	No	72 (85.7)	116 (87.2)	0.838	13 (81.2)	116 (87.2)	0.453	72 (85.7)	13 (81.2)	0.704
	Yes	12 (14.3)	17 (12.8)		3 (18.8)	17 (12.8)		12 (14.3)	3 (18.8)	
Necrosis	No	74 (88.1)	110 (82.7)	0.335	12 (75.0)	110 (82.7)	0.492	74 (88.1)	12 (75.0)	0.231
	Yes	10 (11.9)	23 (17.3)		4 (25.0)	23 (17.3)		10 (11.9)	4 (25.0)	

*P<0.05; **P<0.01; ***P<0.001.

#Intestines, peritoneum, pelvic cavity, abdominal cavity, esophagus and adrenal gland.

In the current cohort (233 patients), the point mutations (84, 36.1%) were most frequently detected in exon 11, followed by deletions (78, 33.5%), compound mutations (55, 23.6%), insertions (10, 4.3%) and tandem duplication mutations (6, 2.6%). Out of the 84 patients with a point mutation, 55 patients’ tumors were located at the stomach, 26 in the small intestine, and 3 in other locations. Out of 78 patients with deletions, 44 patients had a tumor in the stomach, 29 in the intestine, and five were in other sites. Similarly, in patients with compound mutations, 24, 23, and 8 GISTs were located at the stomach, intestine, and other areas, respectively. While patients with insertion mutations had tumors in the stomach. In addition, of the tandem duplication mutations, 4, 1, and 1 GISTs were situated in the stomach, intestine, and other locations in GI, respectively.

Based on the twelve factors (including age, sex, tumor location, tumor diameter, recurrence risk, CD117, DOG-1, CD34, ulcer, mitotic count, bleeding, and necrosis), the differences in distribution among the mutations by merging the five mutation types into three groups (point mutations, deletion/compound mutations and insertion/tandem duplication mutations) were compared subsequently. The results of the comparisons of the three groups are shown in **Table 2**. The point mutations showed no significant difference in deletion/compound mutations (P>0.05). Still, they revealed the age differences (<60: 45.2% vs. 60.2%, P=0.032), tumor location (stomach: 65.5% vs. 51.1%, *P=0.038*), mitotic count (≤5: 58.3% vs. 34.6%, *P=0.001*) and recurrence risk (low and intermediate-risk: 58.1% vs. 36.1%, *P=0.003*). Nevertheless, the distribution of insertion/tandem duplication mutations was similar with that of deletion/compound mutations. Still, it showed significant differences in tumor location (stomach: 87.5% vs. 51.1%, *P=0.006*), mitotic count (≤5: 62.5% vs. 34.6%, *P=0.029*) and recurrence risk (low and intermediate-risk: 75.0% vs. 36.1%, *P=0.003*).

### Mutation landscape and characteristics of exon 11

**Figure 1:**
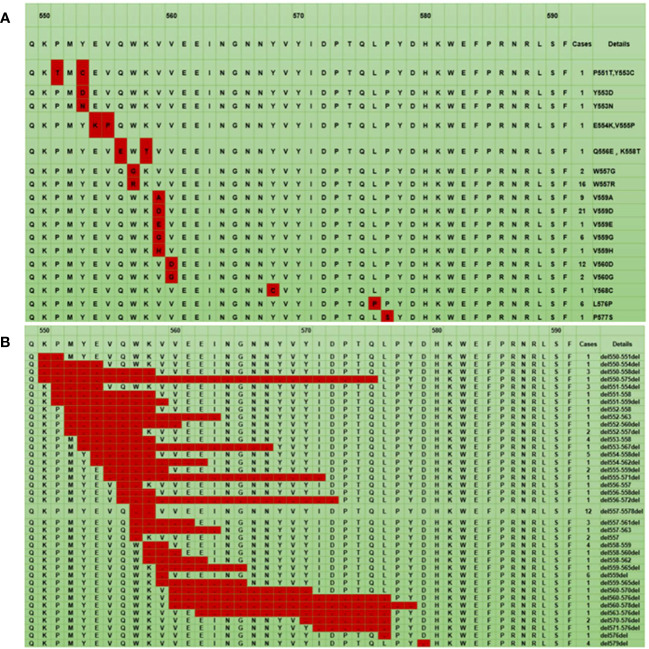
The mutation landscape of KIT exon 11: The red color represents a point mutation **(A)**, The red color represents a deletion mutation **(B)**. * The first line represents the codon site, the second line is the corresponding wild-type amino acid sequence, and the remaining lines are the different mutant amino acid sequences.

The mutation landscape of the 84 points mutations in KIT exon 11 is shown in **Figure 1A**. Most of the *KIT* exon 11 points mutations were located at codons 557, 559 and 560 with the frequency of 22.6% (19/84), 53.6% (45/84), and 16.7% (14/84), respectively, resulting in the amino acid substitutions V559D (21/84, 25.0%), W557R (16/84, 19.0%), V560D (12/84, 14.3%), and L576P (6/84, 7.1%). Additionally, we detected some novel variants (Y553D, Y553N, V559E, V559G, V559H and P577S) in the gene.

For all deletion mutations, the most common sites were in codons 552-560 of exon 11, a hotspot region in the juxtamembrane domain. The codons 557/558 deletion occurred with the highest frequency (15.4%, 12/78) (**Figure 1B**).

**Figure 2:**
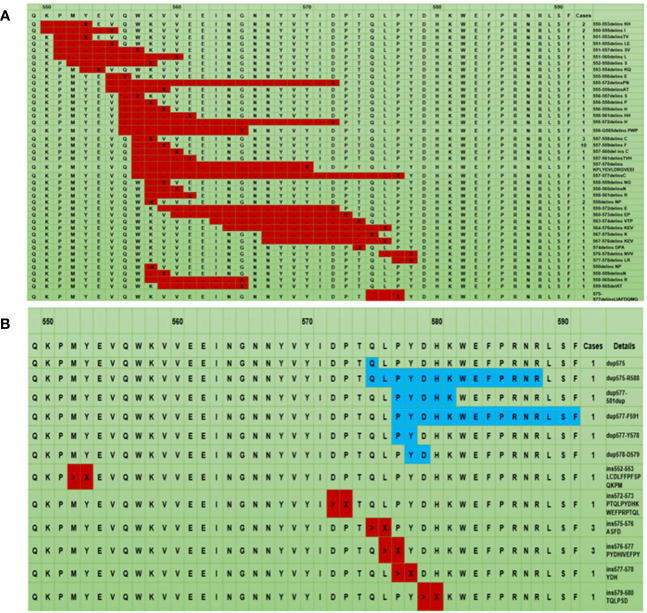
The mutation landscape of KIT exon 11: The black point represents codon deletion, X represents codon insertion, which is an uncertain codon and ranges from 1 to 19 **(A)**, > represents insertion mutation, X represents different codons ranging from 2 to 18. The blue color represents tandem duplication mutation. **(B)**. * The first line represents the codon site, the second line is the corresponding wild-type amino acid sequence, and the remaining lines are the different mutant amino acid sequences.

The compound mutations were polytropic, which combined single-point mutations and multiple insertions. These mutations were in codons 557-559 (18.2%, 10/55) (**Figure 2A**). Most insertion and tandem duplication mutations occurred at codons 570-590 (93.8%, 15/16) (**Figure 2B**).

### Association analysis between mutation status, baseline characteristics and prognosis

**Figure 3:**
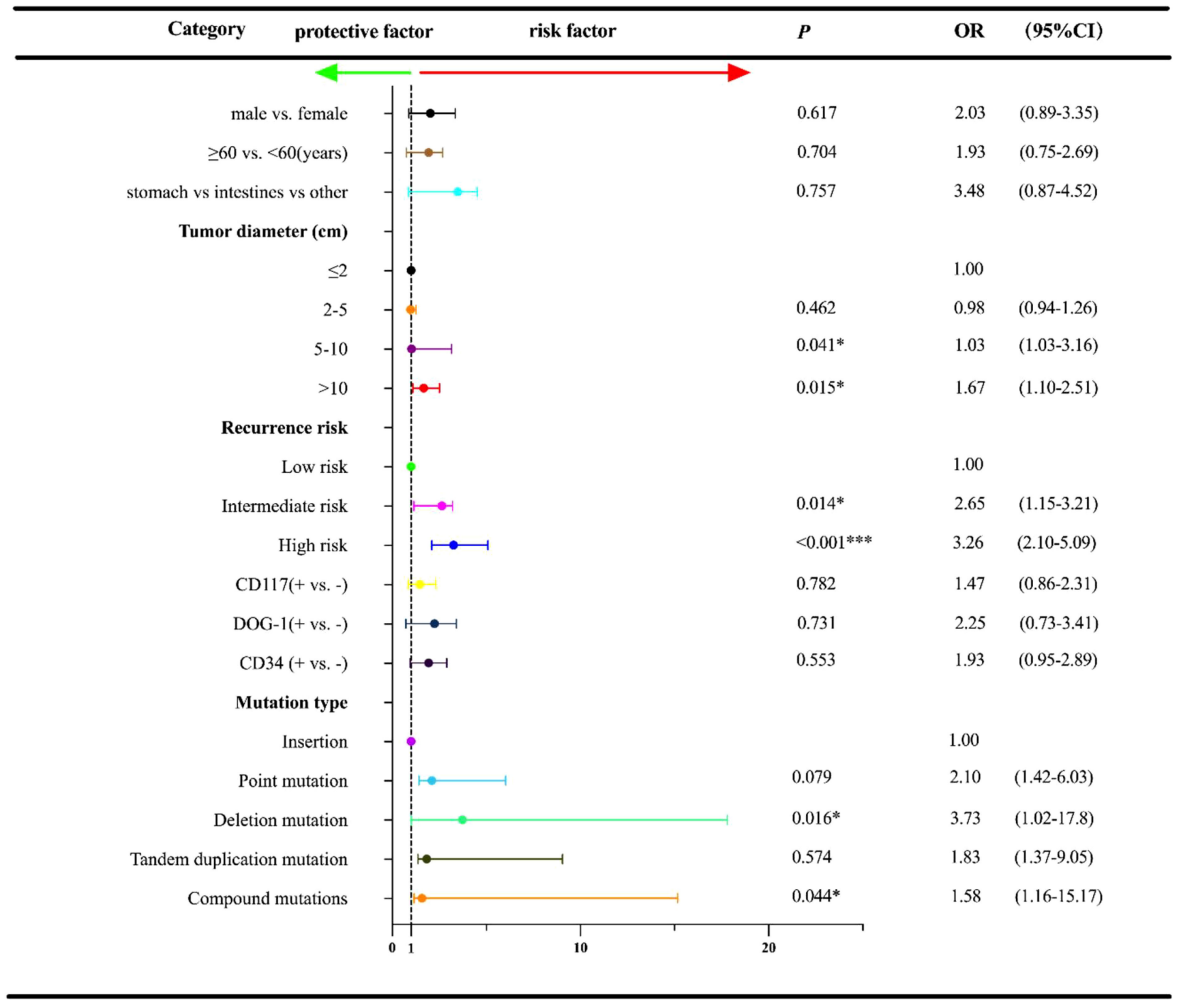
Univariate regression analysis for RFS† according to other characteristics and exon 11 mutation type in the 233 GISTs patients. †RFS, relapse-free survival; OR, Odds ratios; CI, confidence interval; *P<0.05; ***P<0.001.

**Figure 4:**
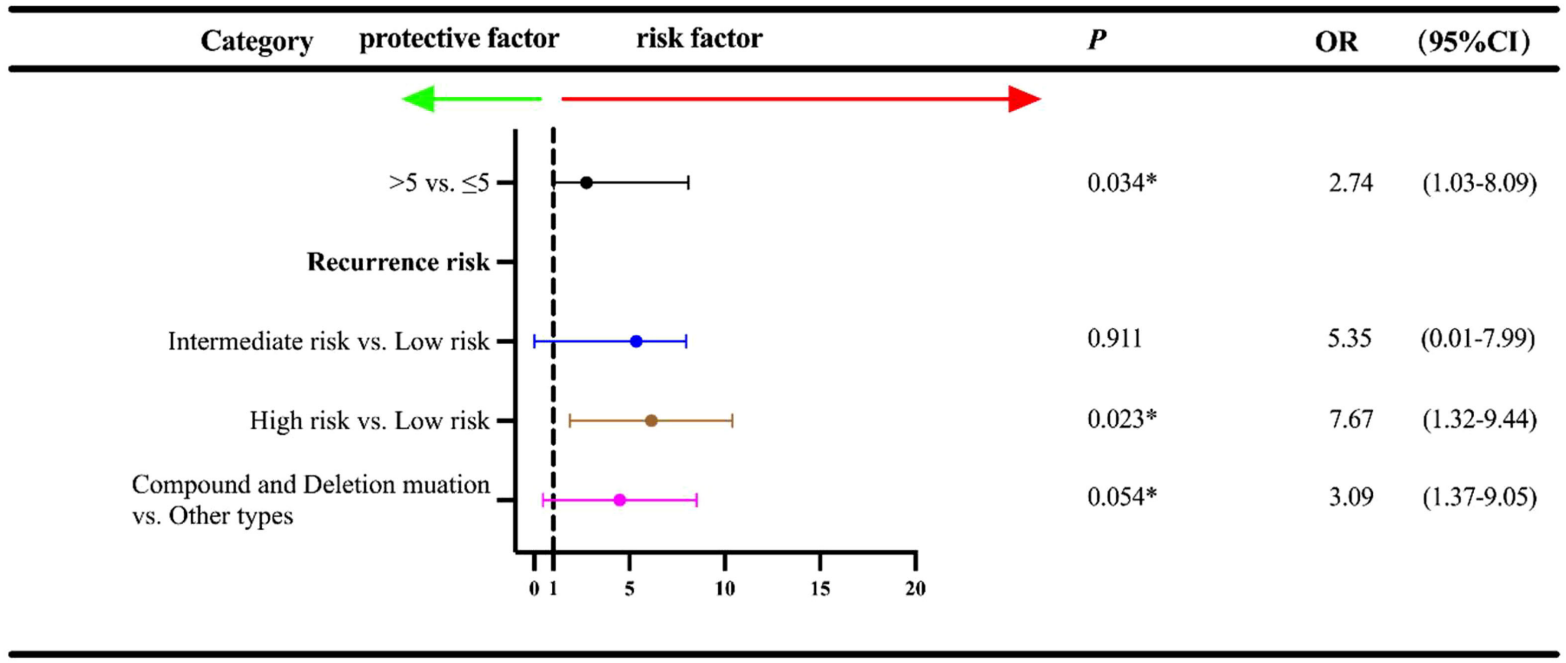
Multivariable regression analysis for RFS among the 233 patients with GISTs.

The date of cutoff data of this study was June 15, 2022, and the median follow-up duration was 28.0 months (range: 3-65 months). At the cutoff date, a total of 23 patients experienced the disease recurrence, and a total of 196 patients underwent follow-up regularly. Univariate analysis was used to identify the association between baseline characteristics and RFS (**Figure 3**). Tumor diameter of >5cm exhibited an inferior prognosis than those with a tumor diameter of ≤2cm (5-10 cm vs. ≤ 2 cm: OR=1.03, 95% CI:1.03-3.16, *P*=0.041; >10 cm vs. ≤ 2 cm: OR=1.67, 95% CI: 1.10-2.51, *P*=0.015). Additionally, patients with the intermediate and high recurrence risk also conferred a worse prognosis compared with that of those with the low risk (intermediate risk vs. low risk: OR=2.65, 95% CI: 1.15-3.21, *P*=0.014; high risk vs. low risk: OR=3.26, 95% CI: 2.10-5.09, *P*<0.001). Of the exon 11 mutation types, deletions and compound mutations were associated with worse prognosis (OR=3.73, 95% CI: 1.02-17.80, *P*=0.016; OR=1.58, 95% CI: 1.16-15.17, *P*=0.044). However, we failed to identify the independent influence of deletions/compound mutations on RFS (OR=3.09, P=0.054), unlike recurrence risk (OR=7.67, P=0.023) and tumor diameter (OR=2.74, P=0.034), which indicated that high recurrence risk and tumor diameter ≥5cm were independent adverse prognostic factors for RFS (**Figure 4**).

**Figure 5:**
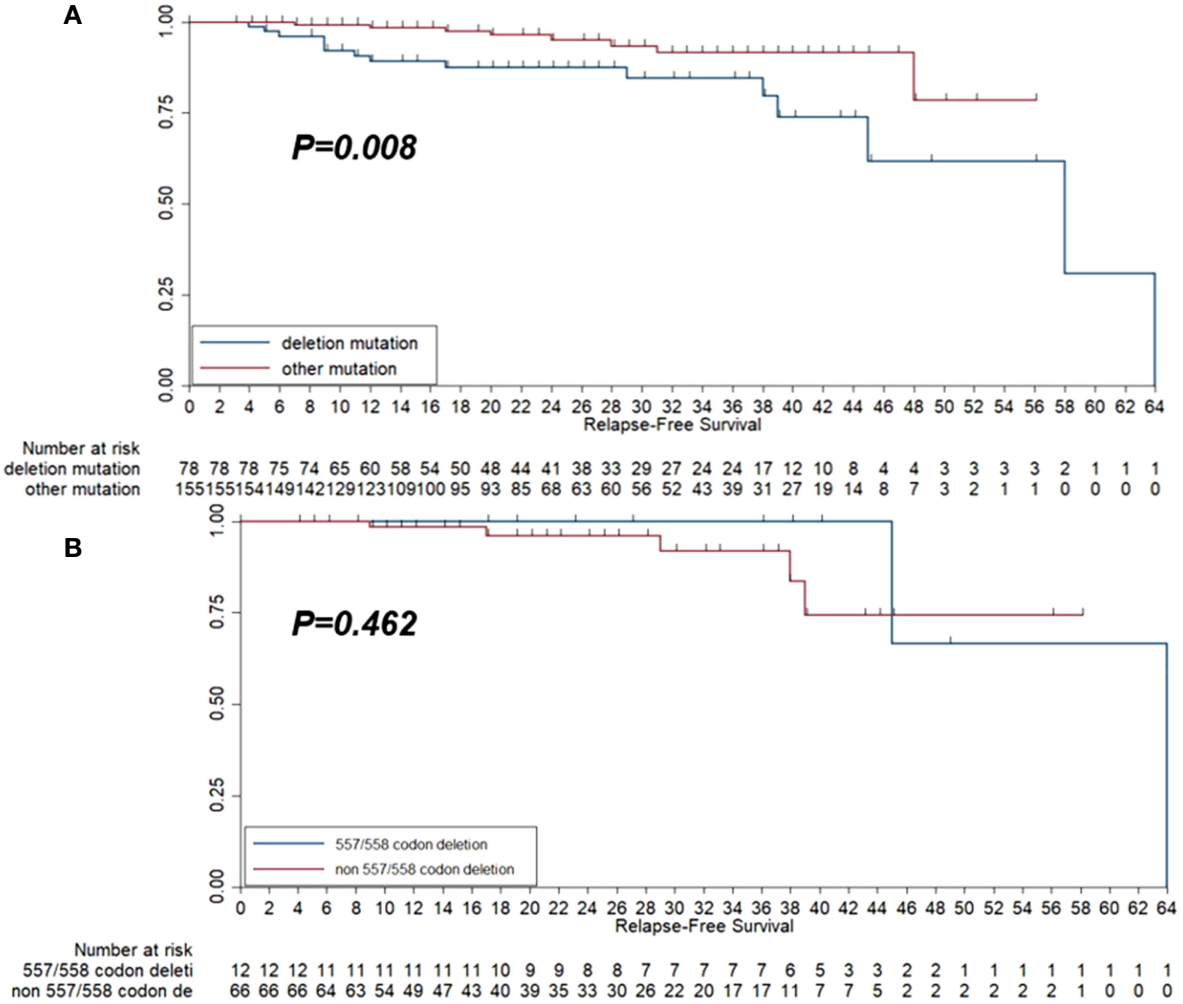
Kaplan-Meier survival curve of relapse-free survival (RFS) among the 233 patients with GISTs according to deletion mutation group and other mutation groups **(A)** and the RFS among the 78 patients who harbored deletion mutation according to 557/558 codon deletion status **(B)**.

Kaplan–Meier survival analysis was adopted to present the survival difference between the deletion/compound mutation group and the other group. As illustrated in **Figure 5A**, the deletion/compound mutation subtype conferred a worse RFS than patients with different mutation subtypes (P=0.008). Furthermore, as exhibited in **Figure 5B**, no difference in RFS between the 557/558 deletions and the other deletions groups was observed (P=0.462).

## Discussion

Studies over the past two decades have demonstrated that approximately 75%-88% of GISTs harbor mutations in the KIT gene and that different types of mutations in the KIT gene are associated with distinct roles in the development of these tumors (20, 21). The present study included a total of 233 GISTs who had exon 11 mutations in the KIT gene to identify the potential significance. The results revealed that the clinicopathological characteristics of GISTs with exon 11 mutations in the KIT gene were similar with those in other previous studies (22, 23).

To the best of our knowledge, although NIH and Armed Forces Institute of Pathology (AFIP) standards were widely recommended for patients with GIST (24), neither considered tumor mutation status for risk stratification. A retrospective analysis of the prognosis in 1765 GISTs concluded that KIT expression was detected in 91% of the patients and that intratumor necrosis, ulcer, and mucosal infiltration were adverse factors (25). Interestingly, our study found that tumor diameter, high risk of recurrence and deletions/compound mutations were poor prognostic factors for RFS in patients with exon 11 mutations. The prognosis of patients with these mutations was heterogeneous. We further confirmed that the point mutations mainly occurred in stomach tumors and had an intermediate risk of recurrence. However, the deletion/compound mutations were more common in tumors in non-stomach locations and had a high risk of recurrence.

Recent studies have found that exon 11 mutations in the KIT gene showed diversity and heterogeneity (26, 27), which included point mutations, deletions, compound mutations (deletion/insertion), insertions and tandem duplication mutations (28). Noteworthily, a retrospective study suggested that the mutations in KIT exon 11 were related to multiple factors, including age, gender, primary tumor location, tumor diameter, mitotic count and CD34 positivity (29). As mentioned above, our results found that five mutation types in exon 11 showed different distributions in twelve factors. Simultaneously, the different KIT exon 11 mutations such as point mutations, compound mutations and deletion mutations were not different based on gender, age, tumor location, CD117, CD34, DOG-1, bleeding or necrosis. However, clinical characteristics such as tumor diameter and the risk of recurrence presented significant differences in the distribution of the mutations. Furthermore, the proportion of different mutation types in exon 11 of the KIT gene also showed a considerable difference: Kim et al. reported previously that in GISTs with exon 11 mutations, deletions accounted for 54.1%, point mutations for 32.9%, compound mutations for 6.6%, tandem duplication mutations for 4.9% and insertions for 1.6% (17). Additionally, a study by Xu CW et al. investigated that among 56 patients with exon 11 mutations in the KIT, deletions mutation accounted for 55.36%, point mutations accounted for 26.79%, and tandem duplication mutations accounted for 14.29%. Deletions accompanied by point mutations accounted for 3.57%, respectively (30). Furthermore, Richard Quek et al. found that the prevalence of deletions mutations, and non-deletions mutations was 42% and 18%, respectively (28). Compared with previous studies, our study indicated that point mutations (36.1%) were most frequently detected in exon 11, followed by deletions (33.5%), compound mutations (23.6%), insertions (4.3%) and tandem duplication mutations (2.6%), which might be attributed to the heterogeneity and ethnic diversity of populations. Furthermore, as indicated by a previous study, DNA sequencing could detect KIT mutations better than PCR-SSCP (31).

Noteworthily, our study found that point mutations in KIT exon 11 were mainly focused on codons 550-560 and the sites with the highest frequency of mutation were codons 557, 559 and 560, which was in line with the previous research (28). Concerning the deletion mutation, which had been described as one of the most common exon 11 mutation types in previous research, approximately 66%-87% of deletions were located at codon 552-560, the first hot spot of exon 11 reported in the literature (4, 18). Consistent with these earlier results, our study also confirmed that the 557/558 deletion was the most common, followed by deletions at 559 and 555-558. Currently, compound mutations of exon 11 of the KIT gene are rare in most studies and their prognostic significance for patients with GIST is still unclear.

Interestingly, Lasota J et al. revealed that compound mutations accounted for 0.5% of the exon 11 mutations of the KIT gene in 700 GISTs (19). However, our study found that the proportion of compound mutations in exon 11 was lower than that of deletions. Additionally, codon 557/559 was the site with the highest frequency of diverse mutations, mainly found in tumors in the stomach and intestine and this proportion was balanced between the two organs. We found that insertion and tandem duplication mutations primarily occurred at the 3’-UTR of exon 11 of the KIT gene at codons 570-590 (32). Collectively, these data suggested that tandem duplication mutation was a prognostic factor with a low proportion of 3.6%, which was 3.2% in our study.

In this study, we identified the molecular landscape of exon 11 mutation in KIT among patients with GIST in the Chinese population, which illustrated the specific mutation profile of exon 11 mutation and contributed to the precise diagnosis for patients with GIST in China. The predictive analysis highlighted that tumor diameter ≥5 cm and deletions/compound mutations might predict worse RFS, which resulted in the further refinement of risk stratification for patients with GISTs who underwent surgical resection. Therefore, for patients with low risk who were concomitant with tumor diameter ≥5 cm and deletions/compound mutations and received surgical resection, adjuvant imatinib treatment might be necessary clinically, according to the conclusion of our study.

Furthermore, we concluded that deletion mutation played an adverse role in RFS compared with the other mutation types, which was in concordance with the previous work of Jiang et al. (14). The underlying mechanisms may be further explained by creating a cellular model by mutating the KIT gene. By analyzing the mutation type in exon 11 of the KIT gene, we revealed that the deletions in exon 11 were essential for the worse prognosis of GISTs, which often occurred in the stomach and was associated with a high recurrence risk (28). Furthermore, a study from Spain elucidated that an increased risk of recurrence and 557/558 deletion in exon 11 was an essential predictor of RFS in GISTs after definitive surgery, which might be considered a new strategy for the prediction of GIST-assisted imatinib efficacy after standard surgery (22). Additionally, another three studies also exhibited that the 557/558 deletion in exon 11 might result in a 5-year decrease of RFS similarly (33–35). Therefore, these studies highlighted the clinical significance of the 557/558 deletion in exon 11 of the KIT gene. As a result, we further compared the prognosis between 557/558 and non-557/558 deletion. However, the result failed to identify significant difference in RFS between 557/558 deletion and non557/558 deletion, which the small sample size and the limited follow-up duration might explain.

From the objective view, limitations existed in this study were unavoidably. Firstly, the sample size of the present study was relatively small, and the clinical significance of exon 11 mutation in KIT among patients with GISTs still needed to be evaluated in a larger population. Secondly, this study was designed as a retrospective analysis; some biases might compromise the investigation, and the conclusion should be interpreted cautiously. Still and all, the baseline characteristics and molecular landscape of different mutation types in KIT exon 11 among Chinese patients with GISTs were elucidated in our study, which was of practical significance for the treatment of patients with GISTs in the future and meaningful for clinicians to improve their understanding of the mutation status and prognostic implications of exon 11 mutations in the KIT gene.

## Conclusion

This study provided the detailed molecular landscape of mutations in exon 11 of the KIT gene among GIST patients. This preliminary study suggest that exon 11 mutations in KIT are common in GISTs with intermediate/high recurrence risk especially when the diameter of the tumor is ≥5. cm. In addition, our study also deduce that deletions/compound mutations may be associated with a worse prognosis. However, further studies including prospective multi-central randomized clinical trial are needed to verify the results shown in this study. There is also a need to investigate the relationship between the different kit exons 11 and patient’s OS with a long-term follow-up. Finally, exploring the relationship between clinical efficacy of imatinib and patients with different exon 11 mutation subtypes will be useful in guidance for medication for GISTs patients which could be the focus of future research.

## Data availability statement

The datasets presented in this study can be found in online repositories. The names of the repository/repositories and accession number(s) can be found in the article/supplementary material.

## Ethics statement

The studies involving humans were approved by The Clinical Research Ethics Committee of the First Hospital of Zhengzhou University (approved number: 2021-KY-1080-002). The studies were conducted in accordance with the local legislation and institutional requirements. The participants provided their written informed consent to participate in this study.

## Author contributions

RZ: Formal Analysis, Investigation, Methodology, Project administration, Writing – original draft. TA: Investigation, Methodology, Software, Writing – review & editing. ML: Methodology, Software, Validation, Writing – review & editing. YZ: Data curation, Investigation, Methodology, Writing – review & editing. RL: Data curation, Investigation, Methodology, Validation, Writing – review & editing. GJ: Investigation, Methodology, Supervision, Validation, Writing – review & editing. JL: Data curation, Formal Analysis, Investigation, Methodology, Writing – review & editing. XC: Data curation, Investigation, Software, Validation, Writing – review & editing. HZ: Formal Analysis, Investigation, Software, Supervision, Validation, Writing – review & editing.
